# Children's mental and behavioral health, schooling, and socioeconomic characteristics during school closure in France due to COVID-19: the SAPRIS project

**DOI:** 10.1038/s41598-021-01676-7

**Published:** 2021-11-17

**Authors:** Maëva Monnier, Flore Moulin, Xavier Thierry, Stéphanie Vandentorren, Sylvana Côté, Susana Barbosa, Bruno Falissard, Sabine Plancoulaine, Marie-Aline Charles, Thierry Simeon, Bertrand Geay, Laetitia Marchand, Pierre-Yves Ancel, Maria Melchior, Alexandra Rouquette, the SAPRIS study group, Nathalie Bajos, Fabrice Carrat, Pierre-Yves Ancel, Marie-Aline Charles, Florence Jusot, Claude Martin, Laurence Meyer, Ariane Pailhé, Alexandra Rouquette, Gianluca Severi, Alexis Spire, Mathilde Touvier, Marie Zins, Cédric Galera

**Affiliations:** 1 Bordeaux Population Health Research Center, INSERM U 1219, Université de Bordeaux, 146 rue Léo Saignat, 33077 Bordeaux Cedex, France; 2 Department of Child and Adolescent Psychiatry, Centre Hospitalier Charles Perrens, Bordeaux, France; 3 ELFE Joint Unit, French Institute for Demographic Studies (Ined), French Institute for Medical Research and Health (Inserm), Paris, France; 4 Inserm, UMR 1219, Vintage Team, Université de Bordeaux, 33000 Bordeaux, France; 5 Santé Publique France, French National Public Health Agency, 94415 Saint-Maurice, France; 6 Department of Social and Preventive Medicine, University of Montreal, Montreal, Canada; 7 Research Unit on Children’s Psychosocial Maladjustment, Montreal, QC Canada; 8 Centre National de la Recherche Scientifique, Institut de Pharmacologie Moléculaire et Cellulaire, Université Côte d’Azur, Valbonne, France; 9 Inserm, UVSQ, CESP, DevPsy, Paris-Saclay University, Paris, France; 10 Centre for Research in Epidemiology and StatisticS (CRESS), Inserm, INRAE, Université de Paris, 75004 Paris, France; 11 Inserm Unité Mixte de Recherche 1153, Obstetrical, Perinatal, and Pediatric Epidemiology, Paris, France; 12 ELFE Joint Unit, Paris, France; 13 EPIPAGE-2 Joint Unit, Paris, France; 14 Research Team (EPOPé), Center for Epidemiology and Statistics, Sorbonne Paris Cité, Département Hospitalo-Universitaire Risks in Pregnancy, Paris Descartes University, Paris, France; 15 Unité de Recherche Clinique-Centre d’Investigations Cliniques P1419, Département Hospitalo-Universitaire Risks in Pregnancy, Cochin Hotel-Dieu Hospital, Paris, France; 16 INSERM, Institut Pierre Louis d’Epidémiologie et de Santé Publique (IPLESP), Equipe de Recherche en Epidémiologie Sociale (ERES), Sorbonne Université, Paris, France; 17 Public Health and Epidemiology Department, AP-HP Paris-Saclay, Bicêtre Hospital, Le Kremlin-Bicêtre, France; 18 Institut de Recherche Interdisciplinaire sur les enjeux Sociaux - Sciences Sociales, Politique, Santé, IRIS (UMR 8156 CNRS - EHESS - U997 INSERM), Aubervilliers, France; 19 INSERM, Institut Pierre Louis d’Epidémiologie et de Santé Publique (IPLESP), Sorbonne Université, Paris, France; 20 Paris-Dauphine University-PSL, Paris, France; 21 French National Centre for Scientific Research, Paris, France; 22 University of Paris Sud, Paris, France; 23 National Institute for Demographic Studies, Paris, France; 24 INSERM, E4N & EN3-Cohorts, Paris, France; 25 Sorbonne Paris Cité Epidemiology and Statistics Research Center (CRESS), Inserm U1153, Inra U1125, Cnam, Paris 13 University, Nutritional Epidemiology Research Team (EREN), Bobigny, France; 26 Sorbonne University, Paris, France

**Keywords:** Psychology, Medical research, Risk factors

## Abstract

COVID-19 limitation strategies have led to widespread school closures around the world. The present study reports children’s mental health and associated factors during the COVID-19 school closure in France in the spring of 2020. We conducted a cross-sectional analysis using data from the SAPRIS project set up during the COVID-19 pandemic in France. Using multinomial logistic regression models, we estimated associations between children’s mental health, children’s health behaviors, schooling, and socioeconomic characteristics of the children’s families. The sample consisted of 5702 children aged 8–9 years, including 50.2% girls. In multivariate logistic regression models, children’s sleeping difficulties were associated with children’s abnormal symptoms of both hyperactivity-inattention (adjusted Odds Ratio (aOR) 2.05; 95% Confidence Interval 1.70–2.47) and emotional symptoms (aOR 5.34; 95% CI 4.16–6.86). Factors specifically associated with abnormal hyperactivity/inattention were: male sex (aOR 2.29; 95% CI 1.90–2.76), access to specialized care prior to the pandemic and its suspension during school closure (aOR 1.51; 95% CI 1.21–1.88), abnormal emotional symptoms (aOR 4.06; 95% CI 3.11–5.29), being unschooled or schooled with assistance before lockdown (aOR 2.13; 95% CI 1.43–3.17), and tutoring with difficulties or absence of a tutor (aOR 3.25; 95% CI 2.64–3.99; aOR 2.47; 95% CI 1.48–4.11, respectively). Factors associated with children’s emotional symptoms were the following: being born pre-term (aOR 1.34; 95% CI 1.03–1.73), COVID-19 cases among household members (aOR 1.72; 95% CI 1.08–2.73), abnormal symptoms of hyperactivity/inattention (aOR 4.18; 95% CI 3.27–5.34) and modest income (aOR 1.45; 95% CI 1.07–1.96; aOR 1.36; 95% CI 1.01–1.84). Multiple characteristics were associated with elevated levels of symptoms of hyperactivity-inattention and emotional symptoms in children during the period of school closure due to COVID-19. Further studies are needed to help policymakers to balance the pros and cons of closing schools, taking into consideration the educational and psychological consequences for children.

## Introduction

The emergence of COVID-19 in 2020 led many countries to implement strict sanitary measures, many of which resulted in substantial changes in children’s lifestyles for several months. On March 31, 2020, 170 countries temporarily closed schools in an attempt to limit the spread of the new coronavirus responsible for this disease^1^. In France, the first lockdown lasted from March 16 to May 11, 2020, and schools and universities, which offer teaching to 14 million students, closed from the beginning of the lockdown until June 22, 2020^2^. School closures, the absence of usual sports and leisure activities, and a reduction in social contacts may have impacted children’s mental health in an unprecedented manner^3–9^. Children may have been exposed to stressors generated by the media, the fear of contagion, the lack of social interactions with individuals outside the immediate family, and by family members affected with COVID-19^10–12^. Moreover, the COVID-19 crisis may have resulted in socioeconomic difficulties (e.g., financial loss and changes in employment status) and impacted the mental health of adults, which in turn may have had indirect effects on mental health in children^10,13,14^. The potential benefits of closing schools to curb the spread of COVID-19 need to be weighed against the effects on children’s mental health^15^.

Although there is no consensus on the effectiveness of school closures to minimize the spread of COVID-19^15–17^, deleterious short-term effects on children’s mental health have been observed. A Chinese study of 7143 college students found that 24.9% experienced anxiety related to the COVID-19 outbreak^3^. Another Chinese cross-sectional study using an online questionnaire on 359 children and 3254 adolescents showed that 22.3% suffered from depressive symptoms during the outbreak^18^. In addition, an internet-based survey evaluated the behavioral problems of 1264 children using the Strengths and Difficulties Questionnaire (SDQ), in two elementary schools in China during the COVID-19 outbreak^19^. The prevalence was 6.3% for hyperactivity/inattention and 4.7% for emotional symptoms^19^. However, to date the majority of studies on this topic have been carried out in China where the epidemic has been of shorter duration than in other parts of the world^3,4,18,20–22^. In France, a survey that used the SDQ on 432 parents showed that 24.7% of their children had hyperactivity/inattention symptoms and that 7.1% had emotional difficulties during the first lockdown in the spring of 2020^14^. This study based on a small sample of children identified significant correlates of these symptoms with parental mental health or financial difficulties, as well as the child’s sleeping difficulties or high screen time use. A study conducted in the Philippines among 952 college students aged 18–22 years old showed that students from poorer households, who do not own laptops and desktop computers, and have a lesser variety of gadgets, exhibited higher disease-related COVID-19 anxiety^12^. Therefore, social inequalities related to children's health were exacerbated by this unprecedented situation^5^. These studies had several limitations like a small sample size or the non-inclusion of other factors that might affect children's mental health, such as chronic illness and number of hours of study at home per day^23^. Furthermore, while a significant number of studies were conducted on the indirect effects of the COVID-19 pandemic on mental health, only a few focused on children^13,24^. More research is therefore needed to quantify the impact of socioeconomic disparities and inequalities that are exacerbated by school closure and that of the lockdown on children's mental health^25^.

The present study sought to assess the factors associated with the mental health of children aged 8–9 years-old during the school closure in the first lockdown in spring of 2020 in France. We focused on children’s hyperactivity/inattention and emotional symptoms, and on a range of factors such as health behaviors (e.g., sleeping difficulties), schooling (e.g., tutoring, time devoted to schoolwork), and socioeconomic characteristics of children’s families (e.g., financial difficulties, type of housing, occupational category).

## Methods

In the early weeks of the COVID-19 crisis, the SAPRIS survey was set up to study the health, social interactions and socioeconomic characteristics of the general population during the crisis in France^26^. The project was based on the collection of data using the same questionnaire on the participants of five large ongoing French cohorts, three of adults (Constances, E3N-E4N and NutriNet-Santé) and two of children (ELFE/EPIPAGE-2). For the present study, we used data collected on the 5702 children aged 8–9 years old in 2020 and participating in the ELFE and EPIPAGE-2 population-based birth cohorts that focus on child health and include late pre-term and term (ELFE) and very and extremely premature children (EPIPAGE-2) born in 2011^27–29^. More details on ELFE and EPIPAGE 2 are available elsewhere^27–29^. Data for the present study were reported by parents during the first lockdown period (from April 16 to May 4, 2020) or immediately after (from May 5 to June 21, 2020), thus encompassing the whole period of school closure^30^. To make the target populations completely disjointed and complementary, 48 children born before 35 weeks in ELFE were excluded. All methods were performed in accordance with the relevant guidelines and regulations. Ethical approval and written informed consents were obtained from all subjects and/or their legal guardian. The study was approved by the Inserm ethics evaluation committee (no 2020.04.24 bis_20.04.22.74247, 2020 April 27), and the CNIL ((no 920193, 2020 April 30). Representatives of the participants tested and validated the questionnaires, but they did not contribute to other aspects related to the design, conduct, reporting or dissemination of the research.

### Children’s mental health

Symptoms of hyperactivity/inattention and emotional symptoms were ascertained by two subscales of the SDQ, a widely used measure of children’s mental health which has satisfactory psychometric properties^31,32^. These symptoms concerned the 15 days prior to the questionnaire and were reported by parents on a 3-point scale (not true, somewhat true, and certainly true; ranges from 0 to 2) to indicate the extent to which each item applied to their children^33,34^. The following five items were used to assess symptoms of hyperactivity/inattention: *“Restless, overactive, cannot stay still for long”; “Constantly fidgeting or squirming”; “Easily distracted, concentration wanders”; “Thinks things out before acting”; and “Sees tasks through to the end, good attention span”*^33^. The five items used to assess the emotional symptoms were: *“Complains of headache/stomach ache”; “Many worries, often seems worried”; “Often unhappy, down-hearted or tearful”; “Nervous or clingy in new situations, easily loses confidence”; and “Many fears, easily scared”*^32^. From the parents' responses to the five items of each subscale, we calculated scores that ranged from 0 to 10 with cut-offs of the French version of the SDQ^35^. Concerning hyperactivity/inattention, a score ≤ 5 is considered normal, equal to 6 as boundary state (moderate), and > 6 as abnormal. For emotional symptoms, a score ≤ 3 is considered normal, equal to 4 as boundary state (moderate), and > 4 as abnormal.

### Covariates

#### Children’s health behavior

Health behaviors included the following: sleeping difficulties (e.g., difficulty falling asleep, waking up frequently or too early at night without being able to go back to sleep) since the beginning of lockdown (yes: new occurrence, increase, or stability, no: decrease, disappearance or none); time per day spent by child on the following activities: (a) reading, drawing, board games; (b) screen for recreation, (c) social network, (d) physical activity (sports or walks outside; indoor and outdoor physical activity).

#### Children’s schooling

Educational characteristics included the following: school situation before lockdown (normal; with assistance or unschooled); average time devoted to schoolwork per day (none or less than 1 h, 1–3 h, > 3 h); tutoring difficulty (tutoring with difficulty, tutoring without difficulty, no tutoring); average screen time for educational reasons per day. Tutoring refers to the presence of someone who can help the child with homework (e.g., parents, siblings, grandparents etc.). Screen time for educational reasons refers to time spent on television or other screens to follow school or educational programs.

#### Socioeconomic characteristics of children’s families

Socioeconomic characteristics included the following: the parents’ occupational category (executive, intermediate and executive, intermediate and employee, independent, laborer, 2 inactive or only one employee/laborer); change in parents’ work situation (change at least for one parent, no change for either parent); parents’ distance working (neither working, one teleworking and the other not working, at least one working outside, both teleworking); perceived financial situation (affluent and constant income; affluent and declining income; modest and constant income; modest and declining income); type of housing (rural house, urban house, flat with balcony/garden, flat without balcony/garden, other); region classified according to the prevalence of COVID-19 at the beginning of the epidemic (High: Ile de France and Grand Est vs Medium: Bourgogne-Franche-Comté, Auvergne-Rhône-Alpes and Hauts-de-France vs. Low: other regions); number of rooms per inhabitant and if the child lived with both parents or not.

#### Others

Additional data included the following: sex; chronic disease (yes, no); born pre-term (yes, no); children’s access to specialized care prior to the COVID-19 epidemic (e.g., physiotherapist, speech therapist, psychologist, rehabilitation) and its continuation during school closures (yes and pursuit, yes but no pursuit, no); COVID-19 cases among household members (yes, no).

### Statistical analysis

We first described child and family sociodemographic characteristics, (i.e., weighted means with standard deviations for continuous variables; frequency with weighted percentages for categorical variables). Descriptive analyses were conducted after correcting the sample data to be representative of children born in France in 2011^36^. For this purpose, a weighting coefficient was calculated. For more information, see the online supplement.

Before performing multiple imputation, we removed variables with more than 30% missing data (screen time and physical activity) and observations with missing data for both outcomes (hyperactivity/inattention and emotional symptoms). Classification and Regression Tree methods (CART, imputation method for mixed data: both Continuous and Categorical)^37^ for multiple imputation were used to handle missing data. Secondly, to test the association between each covariate and mental health outcomes (i.e., hyperactivity/inattention and emotional symptoms), we used multinomial logistic regressions adjusted on prematurity to estimate Odds Ratios (OR) and 95% confidence intervals (CI). Thirdly, to perform relevant covariate selection, we applied a penalized regression method, Elastic-Net, combining bootstrapping with multiple imputed data^38–40^. We used the variable inclusion probability with a 50% threshold to select variables associated with children’s mental health. Finally, we used multinomial logistic regression to estimate adjusted OR and 95% CI for the association between variables selected by Elastic-Net, prematurity and both mental health outcomes.

Data were analyzed using the *nnet* package in *R (version 3.6.1)* with multinomial logistic regressions specified using the *multinom* function. The *mice* package and its *cart* method were used to perform multiple imputation. Finally, the *caret*, *glmnet*, *doParallel* and *dplyr* packages were applied to implement the Elastic-Net method on imputed data.

## Results

### Sample characteristics

The study included 5702 children (Table 1). In total, 2808 (50.2%) children were females, 1161 (19.4%) had access to specialized care prior to the COVID-19 epidemic, 289 (4.5%) had chronic disease, and 1122 (2.9%) were premature children. COVID-19 cases were reported in 235 (4.4%) households. Concerning the children’s mental health, hyperactivity/inattention scores were abnormal for 672 (13.6%) and moderate for 452 (8.2%) children, respectively. Emotional difficulties scores were abnormal or moderate for 404 (7.5%) and 288 (5.8%) children, respectively. In terms of children’s health behaviors, 39.9% had sleeping difficulties. Children spent an average of 2 h and 20 min reading, drawing, and playing board games per day, and 2 h and 45 min doing physical activities per day. Regarding screen time, children spent an average of 3 h and 10 min per day for recreation, and 5 min on social networks. Only 188 (2.8%) children were unschooled or in school with assistance before the lockdown, 2764 (62.8%) spent between 1 and 3 h per day on schoolwork and an average of 50 min on educational programs per day. 4202 (96.7%) children had a tutor at home to help them with their homework.

**Table 1:** Characteristics of children participating in SAPRIS project in France on weighted data (n = 5702).

	Mean (SD)	n (%)
*Sex*		
Male		2890 (49.8)
Female		2808 (50.2)
*Chronic disease*		
Yes		289 (4.5)
No		5079 (95.5)
*Born pre-term*		
Yes		1122 (2.9)
No		4580 (97.1)
*Access to specialized care prior to COVID-19*		
Yes and pursuit during school closures		151 (2.4)
Yes but no pursuit during school closures		1010 (17.0)
No		4217 (80.6)
*Covid cases in household*		
Yes		235 (4.4)
No		5350 (95.6)
**Children’s mental health**		
*Hyperactivity/inattention*		
Abnormal		672 (13.6)
Boundary state		452 (8.2)
Normal		4046 (78.1)
*Emotional symptoms*		
Abnormal		404 (7.5)
Boundary state		288 (5.8)
Normal		4495 (86.7)
**Children’s health behavior**		
*Sleeping difficulties*		
Yes		2092 (39.8)
No		3175 (60.2)
*Time per day spent (in hours) on:*		
Reading, drawing, board games	2.37 (0.06)	
Screen for recreation	3.15 (0.08)	
Social network	0.09 (0.02)	
Physical activity	2.74 (0.08)	
**Children’s schooling**		
*School situation before lockdown*		
Normal		5080 (97.2)
With assistance or unschooled		188 (2.8)
*Time devoted to schoolwork per day*		
None, less than 1 h or do not know		124 (2.6)
1–3 h		2764 (62.8)
> 3 h		1496 (34.5)
Screen time for educational reasons per day (in hours)	0.83 (0.04)	
*Tutoring difficulty*		
Tutoring with difficulty		1003 (22.5)
Tutoring without difficulty		3199 (74.2)
No tutoring		126 (3.3)

As shown in Table 2, the pandemic situation led to a change in work situation in 1483 (43.9%) households, working from home was implemented in 2371 households (32.6%) and 1813 (41.6%) perceived financial difficulties. Children lived mostly in a house (67.4%) and with their two parents (84.5%). 9.0% and 17.3% of children lived in regions with high or moderate prevalence of COVID, respectively.

**Table 2:** Socioeconomic characteristics of children’s families in SAPRIS project in France on weighted data (n = 5702).

	Mean (SD)	n (%)
**Parents' occupational category**^a^		
Executive		1652 (21.9)
Intermediate and executive		1497 (23.5)
Intermediate and employee		1390 (29.1)
Independent		475 (10.3)
Laborer		347 (9.2)
Both inactive or only one employee/laborer		186 (6.0)
**Change in parents’ work situation**^**b**^		
Change at least for one parent		1483(43.9)
No change for either parent		2763 (56.1)
**Parents’ distance-working**^**c**^		
Neither working		1973 (45.6)
One teleworking, the other not working		2013 (28.1)
At least one working outside		1214 (21.8)
Both teleworking		358 (4.5)
**Perceived financial situation**		
Affluent and constant income		3143 (50.6)
Affluent and declining income		557 (7.9)
Modest and constant income		879 (20.3)
Modest and declining income		934 (21.3)
**Type of housing**		
Rural house		2299 (36.5)
Urban house		2235 (30.9)
Urban flat with balcony/garden		714 (23.3)
Urban flat without balcony/garden		218 (6.6)
Other		84 (2.8)
**Region according to prevalence of COVID-19**^**d**^		
High (IDF, GE)		529 (9.0)
Medium (BFC, ARA, HDF)		1096 (17.3)
Low (Other regions)		3928 (73.7)
Number of rooms per inhabitant	1.20 (0.02)	
**Child lives with both parents**		
Yes		4881 (84.5)
No		821 (15.5)

^a^In the absence of a spouse, the respondent who is executive refers to «intermediate and executive» category; which is intermediate corresponds to «intermediate and employee» category; and which is employee, worker or without profession refers to «both inactive or only one employee/labourer» category.

^b^In the absence of a spouse, the respondent who has undergone a change in work situation during the lockdown refers to «change at least for one parent» category and the one who has not undergone any change to «no change for either parent» category.

^c^In the absence of a spouse, the respondent who is not working is associated with the «neither working» category; the one who teleworks refers to «both teleworking» category; and the respondent who works outside to «at least one working outside» category.

^d^Region classified according to the prevalence of COVID-19 at the beginning of the epidemic (High: Ile de France and Grand Est vs Medium: Bourgogne-Franche-Comté, Auvergne-Rhône-Alpes and Hauts-de-France vs Low: other regions).

### Factors associated with hyperactivity/inattention and/or emotional symptoms

The factors associated with hyperactivity/inattention and/or emotional symptoms when adjusting for prematurity are presented in Tables 3 and 4. The factors associated with an increased risk of both hyperactivity/inattention and emotional symptoms in univariate analysis were the following: having chronic disease, being born pre-term, having access to specialized care prior to the COVID-19 epidemic, having difficulties in sleeping, being schooled with assistance or unschooled before the lockdown, having difficulties with tutoring or not having a tutor, low parents’ dominant socio-professional category, presence of change in the parents' work situation, having modest and declining outcome during the lockdown, and not living with both parents. The factor associated with a lower risk of both hyperactivity/inattention and emotional symptoms adjusted on prematurity was having at least one parent who worked outside.

**Table 3:** Factors associated with hyperactivity/inattention: multinomial logistic regressions adjusted on prematurity (n = 5097).

	Hyperactivity/inattention	
	Abnormal versus normal	Boundary state versus normal
*Sex (Female: ref. group)*		
Male	2.05 [1.73; 2.44]	1.61 [1.32; 1.97]
*Chronic disease (No: ref. group)*		
Yes	3.68 [2.78; 4.88]	1.68 [1.11; 2.55]
*Born pre-term (No: ref. group)*		
Yes	1.71 [1.41; 2.07]	1.44 [1.14; 1.81]
*Access to specialized care prior to COVID-19 (No: ref. group)*		
Yes and pursuit during school closures	2.48 [1.62; 3.81]	2.31 [1.41; 3.77]
Yes but no pursuit during school closures	2.40 [1.99; 2.89]	1.56 [1.22; 1.97]
*Covid cases in household (No: ref. group)*		
Yes	1.03 [0.68; 1.55]	1.14 [0.72; 1.81
**Children’s mental health**		
*Emotional symptoms (Normal: ref. group)*		
Abnormal	5.73 [4.54; 7.24]	1.75 [1.21; 2.53]
Boundary state	2.84 [2.09; 3.85]	2.57 [1.82; 3.62]
**Children’s health behavior**		
*Sleeping difficulties (No: ref. group)*		
Yes	2.68 [2.26; 3.17]	1.88 [1.54; 2.28]
**Children’s schooling**		
*School situation before lockdown (Normal: ref. group)*		
With assistance or unschooled	3.85 [2.74; 5.40]	1.84 [1.12; 3.01
*Time devoted to schoolwork per day (None, less than 1 h: ref. group)*		
1–3 h	0.51 [0.33; 0.81]	0.78 [0.42; 1.47]
> 3 h	0.52 [0.33; 0.82]	0.91 [0.48; 1.73]
*Tutoring (Tutoring without difficulty: ref. group)*		
Tutoring with difficulty	3.92 [3.25; 4.73]	2.29 [1.82; 2.87]
No tutoring	2.96 [1.85; 4.72]	1.95 [1.10; 3.44]
**Socioeconomic characteristics**		
*Parents' occupational category (Executive: ref. group)*		
Intermediate and executive	1.34 [1.06; 1.70]	0.90 [0.68; 1.18]
Intermediate and employee	1.58 [1.24; 2.00]	1.28 [0.99; 1.66]
Independent	1.45 [1.05; 2.01]	0.95 [0.64; 1.42]
Both inactive or only one employee/laborer	1.15 [0.77; 1.71]	1.07 [0.70; 1.65]
Laborer	3.03 [2.05; 4.48]	1.43 [0.84; 2.42]
*Change in parents’ work situation (No change for either parent: ref. group)*		
Change at least for one parent	1.33 [1.11; 1.59]	1.13 [0.91; 1.39]
*Parents’ distance-working (No one working: ref. group)*		
At least one working outside	0.73 [0.60; 0.89]	0.94 [0.74; 1.18]
Both teleworking	0.78 [0.63; 0.98]	0.83 [0.63; 1.09]
One teleworking and the other not working	0.73 [0.51; 1.06]	0.80 [0.52; 1.24]
*Perceived financial situation (Affluent and constant income: ref. group)*		
Affluent and declining income	1.19 [0.89; 1.60]	1.47 [1.07; 2.02]
Modest and constant income	1.52 [1.21; 1.91]	1.29 [0.98; 1.71]
Modest and declining income	2.01 [1.62; 2.49]	1.82 [1.41; 2.35]
*Type of housing (Rural house: ref. group)*		
Urban house	0.77 [0.64; 0.94]	1.07 [0.86; 1.33]
Urban flat with balcony/garden	1.04 [0.80; 1.34]	1.26 [0.93; 1.71]
Urban flat without balcony/garden	1.32 [0.89; 1.94]	0.83 [0.46; 1.49]
Other	1.34 [0.70; 2.56]	2.65 [1.43; 4.91]
*Regions affected by COVID (Low: ref. group)*^*a*^		
High (IDF, GE)	0.93 [0.69; 1.26]	1.21 [0.88; 1.68]
Medium (BFC, ARA, HDF)	0.98 [0.79; 1.21]	1.14 [0.90; 1.46]
Number of rooms per inhabitant	0.80 [0.63; 1.00]	0.96 [0.79; 1.17]
*Child living with both parents (Yes: ref. group)*		
No	1.53 [1.24; 1.90]	1.18 [0.90; 1.55]

^a^Region classified according to prevalence of COVID-19 at beginning of epidemic (High: Ile de France and Grand Est vs Medium: Bourgogne-Franche-Comté, Auvergne-Rhône-Alpes and Hauts-de-France vs Low: other regions).

**Table 4:** Factors associated with emotional symptoms: multinomial logistic regressions adjusted on prematurity (n = 5097).

	Emotional symptoms	
	Abnormal versus normal	Boundary state versus normal
*Sex (Female: ref. group)*		
Male	0.81 [0.66; 1.00]	0.73 [0.57; 0.93]
*Chronic disease (No: ref. group)*		
Yes	2.56 [1.81; 3.63]	2.00 [1.29; 3.11]
*Born pre-term (No: ref. group)*		
Yes	1.62 [1.28; 2.06]	1.25 [0.94; 1.68]
*Access to specialized care prior to COVID-19 (No: ref. group)*		
Yes and pursuit during school closures	2.20 [1.31; 3.68]	2.12 [1.17; 3.84]
Yes but no pursuit during school closures	2.01 [1.59; 2.55]	1.69 [1.28; 2.24]
*Covid cases in household (No: ref. group)*		
Yes	1.66 [1.08; 2.56]	1.18 [0.66; 2.10]
**Children’s health behavior**		
*Sleeping difficulties (No: ref. group)*		
Yes	6.29 [4.93; 8.03]	3.15 [2.46; 4.04]
**Children’s schooling**		
*School situation before lockdown (Normal: ref. group)*		
With assistance or unschooled	1.69 [1.07; 2.68]	1.61 [0.92; 2.81]
*Time devoted to school work per day (None, less than 1 h: ref. group)*		
1–3 h	0.78 [0.41; 1.48]	1.03 [0.45; 2.36]
> 3 h	0.86 [0.45; 1.63]	1.07 [0.47; 2.47]
*Tutoring (Tutoring without difficulty: ref. group)*		
Tutoring with difficulty	1.99 [1.58; 2.52]	2.12 [1.62; 2.78]
No tutoring	1.81 [1.02; 3.22]	1.38 [0.64; 2.98]
**Socioeconomic characteristics**		
*Parents' occupational category (Executive: ref. group)*		
Intermediate and executive	1.72 [1.27; 2.34]	1.21 [0.88; 1.68]
Intermediate and employee	1.73 [1.27; 2.36]	1.23 [0.88; 1.71]
Independent	1.71 [1.13; 2.60]	0.90 [0.54; 1.51]
Both inactive or only one employee/laborer	1.92 [1.22; 3.01]	0.80 [0.43; 1.49]
Laborer	3.23 [1.99; 5.24]	1.66 [0.90; 3.07]
*Change in parents’ work situation (No change for either parent: ref. group)*		
Change at least for one parent	1.46 [1.17; 1.82]	1.13 [0.87; 1.47]
*Parents’ distance-working (neither working: ref. group)*		
At least one working outside	0.73 [0.57; 0.94]	0.99 [0.74; 1.32]
Both telework	0.82 [0.62; 1.09]	0.96 [0.69; 1.34]
One teleworks and the other does not work	0.82 [0.51; 1.29]	1.17 [0.72; 1.90]
*Perceived financial situation (Affluent and constant income: ref. group)*		
Affluent and declining income	0.89 [0.59; 1.34]	1.28 [0.84; 1.93]
Modest and constant income	1.73 [1.32; 2.27]	1.49 [1.06; 2.09]
Modest and declining income	1.96 [1.50; 2.55]	2.14 [1.58; 2.89]
*Type of housing (Rural house: ref. group)*		
Urban house	0.79 [0.63; 1.00]	1.01 [0.76; 1.34]
Urban flat with balcony/garden	0.96 [0.69; 1.34]	1.69 [1.20; 2.38]
Urban flat without balcony/garden	1.62 [1.03; 2.54]	1.43 [0.79; 2.31]
Other	0.97 [0.41; 2.26]	1.36 [0.53; 3.46]
*Regions affected by COVID (Low: ref. group)*^*a*^		
High (IDF, GE)	1.24 [0.88; 1.75]	0.71 [0.45; 1.14]
Medium (BFC, ARA, HDF)	1.11 [0.86; 1.44]	0.75 [0.54; 1.05]
Number of rooms per inhabitant	0.96 [0.77; 1.19]	0.76 [0.54; 1.07]
*Child living with both parents (Yes: ref. group)*		
No	1.35 [1.03; 1.77]	1.18 [0.85; 1.64]

^a^Region classified according to prevalence of COVID-19 at beginning of epidemic (High: Ile de France and Grand Est vs Medium: Bourgogne-Franche-Comté, Auvergne-Rhône-Alpes and Hauts-de-France vs Low: other regions).

Factors exclusively associated with an increased risk of hyperactivity/inattention adjusted on prematurity were the following: being a boy; having emotional symptoms. Factors associated with a decreased risk of symptoms of hyperactivity/inattention were the following: spending 1–3 h or more than 3 h per day devoted to schoolwork, having parents working remotely, and living in an urban environment.

In contrast, factors only associated with an increased risk of emotional symptoms adjusted on prematurity were the following: presence of COVID-19 cases in the household, hyperactivity/inattention symptoms, having modest and constant income, and living in an urban flat without a balcony/garden. The only factor associated with a lower risk of emotional symptoms was being a boy.

Finally, the number of rooms per inhabitant and the French regions most affected by the COVID-19 epidemic were not associated with children’s mental health.

### Factors associated with children’s mental health after Elastic Net selection

The factors most strongly associated with children’s mental health after using Elastic Net selection are presented in Tables 5 and 6. Elastic Net selected 10 factors as most associated with hyperactivity/inattention in children: sex, access to specialized care prior to the COVID-19 epidemic, having emotional symptoms, having sleeping difficulties, being schooled with assistance or unschooled before the lockdown, devoting 1 h or less to school work per day, having difficulties with tutoring or not having a tutor, low parents’ dominant socio-professional category, living in an urban house, and not living with both parents (Table 5).

**Table 5:** Associations between most predictive factors from elastic net selection and hyperactivity/inattention symptoms: multivariate multinomial logistic regressions (n = 5097).

	Hyperactivity/inattention^a^	
	Abnormal versus normal	Boundary state versus normal
*Sex (Female: ref. group)*		
Male	2.29 [1.90; 2.76]	1.75 [1.42; 2.14]
*Access to specialized care prior to COVID-19 (No: ref. group)*		
Yes and pursuit during school closures	1.42 [0.87; 2.32]	1.80 [1.08; 2.99]
Yes but no pursuit during school closures	1.51 [1.21; 1.88]	1.27 [0.99; 1.64]
**Children’s mental health**		
*Emotional symptoms (Normal: ref. group)*		
Abnormal	4.06 [3.11; 5.29]	1.43 [0.97; 2.09]
Boundary state	2.14 [1.54; 2.97]	2.11 [1.47; 3.01]
**Children’s health behavior**		
*Sleeping difficulties (No: ref. group)*		
Yes	2.05 [1.70; 2.47]	1.71 [1.39; 2.10]
**Children’s schooling**		
*School situation before lockdown (Normal: ref. group)*		
With assistance or unschooled	2.13 [1.43; 3.17]	1.24 [0.73; 2.10]
*Time devoted to schoolwork per day (None|less than 1 h: ref. group)*		
1–3 h	0.55 [0.34; 0.91]	0.85 [0.45; 1.60]
> 3 h	0.56 [0.33; 0.92]	0.98 [0.51; 1.87]
*Tutoring (Tutoring without difficulty: ref. group)*		
Tutoring with difficulty	3.25 [2.64; 3.99]	2.04 [1.61; 2.57]
No tutoring	2.47 [1.48; 4.11]	1.83 [1.02; 3.31]
**Socioeconomic characteristics**		
*Parents' occupational category (Executive: ref. group)*		
Intermediate and executive	1.20 [0.94; 1.55]	0.87 [0.66; 1.14]
Intermediate and employee	1.25 [0.97; 1.62]	1.18 [0.90; 1.55]
Independent	1.26 [0.88; 1.80]	0.92 [0.61; 1.39]
Both inactive or only one employee/laborer	0.89 [0.58; 1.38]	1.01 [0.64; 1.57]
Laborer	1.66 [1.01; 2.71]	1.11 [0.61; 2.03]
*Type of housing (Rural house: ref. group)*		
Urban house	0.79 [0.64; 0.97]	1.06 [0.84; 1.33]
Flat with balcony/garden	0.95 [0.71; 1.26]	1.17 [0.85; 1.60]
Flat without balcony/garden	1.05 [0.67; 1.64]	0.74 [0.40; 1.36]
Other	1.07 [0.53; 2.17]	2.28 [1.20; 4.35]
*Child living with both parents (Yes: ref. group)*		
No	1.21 [0.92; 1.59]	1.06 [0.77; 1.45]

^a^Adjusted on sex, prematurity, access to specialized care prior to COVID-19 and its continuation during school closures, emotional symptoms, sleeping difficulties, school situation before lockdown, time devoted to schoolwork, tutoring, parents’ occupational category, type of housing, and child living with both parents.

**Table 6:** Associations between most predictive factors from elastic net selection and emotional symptoms: multivariate multinomial logistic regressions (n = 5097).

	Emotional symptoms^a^	
	Abnormal versus normal	Boundary state versus normal
*Born pre-term (No: ref. group)*		
Yes	1.34 [1.03; 1.73]	1.13 [0.83; 1.53]
*COVID cases in household (No: ref. group)*		
Yes	1.72 [1.08; 2.73]	1.14 [0.63; 2.05]
**Children’s mental health**		
*Hyperactivity/inattention (Normal: ref. group)*		
Abnormal	4.18 [3.27; 5.34]	2.22 [1.62; 3.04]
Boundary state	1.37 [0.94; 2.00]	2.12 [1.50; 3.01]
**Children’s health behavior**		
*Sleeping difficulties (No: ref. group)*		
Yes	5.34 [4.16; 6.86]	2.79 [2.17; 3.60]
**Socioeconomic characteristics**		
*Parents' occupational category (Executive: ref. group)*		
Intermediate and executive	1.61 [1.16; 2.21]	1.15 [0.82; 1.60]
Intermediate and employee	1.38 [0.97; 1.94]	1.03 [0.72; 1.48]
Independent	1.48 [0.94; 2.34]	0.72 [0.42; 1.23]
Both inactive or only one employee/laborer	1.63 [0.99; 2.66]	0.66 [0.35; 1.27]
Laborer	1.67 [0.97; 2.88]	1.04 [0.54; 2.01]
*Change in parents’ work situation (No change for either parent: ref. group)*		
Change at least for one parent	1.17 [0.91; 1.51]	0.93 [0.70; 1.25]
*Perceived financial situation (Affluent and constant income: ref. group)*		
Affluent and declining income	0.79 [0.51; 1.22]	1.29 [0.84; 1.98]
Modest and constant income	1.45 [1.07; 1.96]	1.51 [1.05; 2.15]
Modest and declining income	1.36 [1.01; 1.84]	2.03 [1.45; 2.84]

^a^Adjusted on prematurity, COVID cases in household, hyperactivity/inattention symptoms, sleeping difficulties, parents’ occupational category, change in parents’ work situation and perceived financial situation.

After adjusting for these 10 factors most associated with hyperactivity/inattention symptoms and prematurity, boys remained at greater risk for abnormal hyperactivity-inattention than girls (aOR 2.29; 95% CI 1.90–2.76). Children who had regular care before the COVID-19 epidemic and could not continue the sessions during the lockdown had an increased risk of abnormal hyperactivity/inattention symptoms (aOR 1.51; 95% CI 1.21–1.88).

Concerning children’s mental health, compared to normal children, children with abnormal emotional symptoms or at boundary state were associated with higher risk of abnormal hyperactivity/inattention symptoms (aOR 4.06; 95% CI 3.11–5.29 and aOR 2.14; 95% CI 1.54–2.97, respectively). Regarding the children’s health behavior, risk of abnormal hyperactivity/inattention symptoms or at boundary state appeared to be higher for children with sleeping difficulties (OR 2.05; 95% CI 1.70–2.47, OR 1.71; 95% CI 1.39–2.10) than in those without sleeping difficulties (Table 5).

Regarding children’s schooling, being schooled with assistance or unschooled was associated with a higher risk of abnormal hyperactivity/inattention (aOR 2.13; 95% CI 1.43–3.17). Spending 1–3 h per day devoted to schoolwork or more than 3 h seemed to be associated with less risk of abnormal hyperactivity/inattention symptoms than spending no time or less than 1 h (aOR 0.55; 95% CI 0.34–0.91 and aOR 0.56; 95% CI 0.33–0.92, respectively). In this multivariate analysis, having difficulties with tutoring or not having a tutor were still associated with a higher risk of abnormal hyperactivity/inattention (aOR 3.25; 95% CI 2.64–3.99 and aOR 2.47; 95% CI 1.48–4.11, respectively).

For socioeconomic characteristics, the low parents’ occupational category (laborer) was still associated with a higher risk of abnormal hyperactivity/inattention as compared to the executive category (aOR 1.66; 95% CI 1.01–2.71). Compared to living in a rural house, living in an urban house was associated with less risk of abnormal hyperactivity/inattention symptoms (aOR 0.79; 95% CI 0.64–0.97).

Concerning the factors most associated with emotional symptoms, Elastic Net selected the following seven variables: being born pre-term, COVID-19 cases in the household, hyperactivity-inattention symptoms, sleeping difficulties, parents’ occupational category, change in parents’ work situation, and financial situation (Table 6). The results described below are adjusted on these seven variables.

Children born pre-term remained at a higher risk of abnormal emotional symptoms (aOR 1.34; 95% CI 1.03–1.73). The presence of COVID-19 cases in the household (aOR 1.72; 95% CI 1.08–2.73), abnormal symptoms of hyperactivity/inattention (aOR 4.18; 95% CI 3.27–5.34) and sleeping difficulties (aOR 5.34; 95% CI 4.16–6.86) remained risk factors of abnormal emotional symptoms (Table 6). In this multivariate analysis, only the “intermediate and executive” parents’ occupational category was still associated with a higher risk of abnormal emotional symptoms as compared to the "executive" category (aOR 1.61; 95% CI 1.16–2.21). Having a modest income remained a risk factor of abnormal emotional symptoms (aOR 1.45; 95% CI 1.07–1.96; aOR 1.36; 95% CI 1.01–1.84). A change in parents’ work situation during school closures was no longer significantly associated with emotional symptoms.

## Discussion

### Main findings and interpretations

Our study showed high rates of hyperactivity/inattention and emotional symptoms during school closure due to COVID-19 in France. It also helped in identifying risk factors of hyperactivity/inattention and/or emotional symptoms in children. Difficulties in sleeping and parents’ occupational category were the only factors independently associated in the multivariate models with both dimensions of mental disorder. Factors specifically associated with hyperactivity/inattention were the following: sex, presence of regular care and its pursuit during school closure, emotional symptoms, school situation, presence of tutoring and difficulties with it, and type of housing. Factors related to emotional symptoms were the following: being born pre-term, the presence of COVID-19 cases in the household, hyperactivity/inattention and financial difficulties.

### Children’s sex, chronic disease, specialized care and being born pre-term

In the context of the COVID-19 crisis, our results indicate a higher risk of hyperactivity/inattention among boys than girls, and a higher risk of emotional symptoms among girls than boys. Findings by others were similar outside the context of the COVID-19 pandemic^41,42^ and our findings are consistent with those of recent studies on it^4,43,44^. A cross-sectional online survey of 8079 Chinese adolescents aged 12–18 affected by the COVID-19 pandemic revealed that female gender was the most important risk factor for depressive and anxiety symptoms^4^.

After adjustment on covariates, we found that children who had access to specialized care prior to the COVID-19 pandemic and who were unable to continue follow up during school closures had a higher risk of abnormal hyperactivity-inattention. Being born pre-term was associated with a higher risk of abnormal emotional symptoms during the COVID-19 pandemic. These results are coherent with epidemiological studies conducted outside the COVID-19 context which found that pre-and perinatal factors (e.g. prematurity, chronic disease) are associated with Attention-Deficit/Hyperactivity Disorder (ADHD)^45–47^ and emotional problems^48,49^, although the definite causes remain unknown. While being born pre-term is a known risk factor for hyperactivity/inattention disorder^50,51^, it is the association between prematurity and emotional symptoms that is emphasized by our findings.

### COVID-19 cases in the household

In our multivariate analyses, the presence of COVID-19 cases in the household seemed to be a risk factor of abnormal emotional symptoms in children. This was one of the strongest associations according to Elastic Net. These results are in line with a large cross-sectional online survey of 44,447 college students conducted in China^52^. Compared with students who reported not having infected or suspected cases in family members and relatives, those who reported having confirmed and suspected cases in family members and relatives had a higher risk of depressive symptoms (OR 4.06; 95% CI 1.62–10.19 and OR 2.11; 95% CI 1.11–4.00, respectively)^52^. Interestingly, several studies evidenced an association between emotional symptoms and COVID-19 epidemic-related factors such as potential exposure to the virus, fear of infection (i.e., self- and/or family members), and loss of loved ones^3,53,54^. Other studies found an increased risk of post-traumatic stress disorder (PTSD) symptoms^55^ and moderate to high perceived stress scores^56^ in isolated/quarantined individuals during the COVID-19 pandemic. Mental stress, anxiety, depressive symptoms, insomnia and fear are triggered by the pandemic itself, mandatory preventive measures, and COVID-19-related circumstances^53,54,57^.

### Children’s mental health

Our findings showed that during the school closure in France, hyperactivity/inattention and emotional symptoms were associated. Elastic Net found this to be one of the strongest associations. Our results confirm those of another study conducted outside the epidemic context which showed a stronger correlation between hyperactivity/inattention and emotional symptoms in the SDQ^34^.

### Children’s health behavior: sleep difficulties

Our study is consistent with recent reports on the association between difficulties in sleeping and children's mental health during the COVID-19 pandemic^14^, in particular with hyperactivity/inattention and emotional symptoms. Difficulty in sleeping is one of the most predictive variables of hyperactivity/inattention in children, as well as emotional symptoms. Our findings confirm those of previous studies conducted outside the COVID-19 pandemic^58–60^. Hyperactivity/inattention and emotional symptoms are both characteristics of children with ADHD. A meta-analysis of 16 studies comparing sleep in ADHD children versus controls found that the former were significantly more impaired than the latter on most subjective and some objective sleep measures^60^. The relationship between ADHD and difficulties in sleeping is bidirectional^59,61–63^. ADHD may cause sleep problems and sleep problems may cause or mimic ADHD^58,59^. Moreover, a reciprocal relationship between sleep quality and anxiety problems seems very likely, although the specific mechanisms that contribute to these sleep disturbances remain unclear^64–67^.

### Children’s schooling: school situation, time devoted to schoolwork, and tutoring

In multivariate analyses, being unschooled or schooled with assistance before the lockdown was associated with a higher risk of abnormal hyperactivity/inattention. Spending 1–3 h or more than 3 h per day on schoolwork seemed to be associated with a lower risk of abnormal hyperactivity/inattention symptoms than spending no time or less than 1 h. Furthermore, children without a tutor and those with tutoring difficulties were at greater risk for abnormal hyperactivity–inattention compared with those without tutoring difficulties. Our results are consistent with those of a previous study, which found that prolonged school closure regardless of the epidemic context can cause significant mental health problems in children, especially among economically disadvantaged groups^68^. While schools are essential for children’s academic education, they also play an important role in addressing their physiological and mental health needs^68,69^. However, according to Elastic Net, these schooling variables are not strongly associated with emotional symptoms.

### Socioeconomic characteristics of children’s families

Our multivariate analyses found an increased risk of abnormal emotional symptoms in children whose families experienced financial difficulties. Children of parents in a low socio-professional category were also the most affected in terms of hyperactivity/inattention. Similar results were recently reported in a French community-based sample of 432 parents and their children during the COVID-19 lockdown^14^. The authors showed that family socioeconomic factors such as financial difficulties and unemployed parents were significantly associated with a higher risk of children’s mental health problems, such as hyperactivity/inattention and emotional symptoms^14^. Stability of family income was significantly associated with children's psychological difficulties during the COVID 19 pandemic^3^, due to economic decline and the stress that it causes^70,71^.

Our adjusted analysis showed that, compared to living in a rural house, living in an urban house was associated with a lower risk of hyperactivity/inattention during school closure in France. The type of housing was not associated with emotional symptoms in multivariate analysis after an Elastic Net selection. These results are contrary to most studies showing a lower risk of ADHD in children living in rural rather than urban areas^72,73^. A large web-based survey of 8177 Italian students found that poor housing (i.e., living in apartments < 60 m^2^ with poor views and poor indoor quality) was associated with an increased risk of depressive symptoms during lockdown^74^. In addition, an Iranian study which evaluated people's preferences and priorities to choose healthy homes after the COVID-19 pandemic revealed that the most critical priorities for residents were natural light, having a view, acoustic protection, and an open or semi-open space^75^. Our conflicting results may be explained by the greater proximity to other children in urban than in rural areas.

Contrary to findings in the literature^18^, our adjusted analysis did not find a significant association between living in one of the regions of France most affected by COVID 19 and children's mental health. Finally, living with both parents was found to be protective for hyperactivity/inattention but not for emotional symptoms. Living with both parents has been shown to be a protective factor for children's mental health in the context of the COVID-19 pandemic, since a Chinese study of 7143 college students found that living with parents was a protective factor against anxiety (OR 0.75; 95% CI 0.60–0.95)^3^. A recent study on the mental health of children aged 9–18 in France also showed the protective effect of a two-parent family on the psychological distress of children during the COVID-19 pandemic^76^.

### Strengths and limitations

This study has some limitations. First, owing to too many missing data regarding screen time and physical activity, we were unable to estimate the associations between these variables and the presence of hyperactivity/inattention or emotional symptoms in children. The results could be different or even more marked in the general population. Second, only a few parents reported their alcohol and smoking consumption as well as their mental disorders, which prevented us from taking these variables into account in our analyses. Third, we did not have any objective measures of sleep quality but only a parent's self-reported measure of difficulty sleeping. Fourth, we had no data on the consumption of media information related to the pandemic in minutes per day and quality of information received. We did not have access to the children's temperament which has already been shown to be a predictor of emotional disorders and hyperactivity/inattention in children^77^. It could be interesting for future research to take this variable into account, especially since there is already a validated tool in French^78^. Therefore, we were not able to study the association between these environmental factors with children's mental health, even though significant associations have already been reported^44,53,79^. Fifth, our data were collected during a period of school closure in the first COVID-19 wave in France, so we could not analyze data on children's mental health prior to the lockdown. As a result, we were unable to assess the impact of school closure on children's mental health independently from that of the COVID-19 pandemic. Sixth, we could not re-evaluate the mental health of the children, yet it is possible that the reopening of schools in France has resulted in health benefits for them. Future study designs should include re-assessment of mental health and compare children's health scores between countries that implemented vs. did not implement school closure as a preventive measure. Future research should also investigate potential environmental factors that might influence children's mental health, such as parental history of mental illness, consumption of media information related to COVID-19, and domestic violence. In addition, the causal relationship between environmental factors related to COVID-19 and children’s mental health should be explored.

The study also has some strengths. First, it includes the large number of parent respondents during school closures in France at the beginning of the COVID 19 pandemic. It also includes pre-term children, a population more at risk for hyperactivity/inattention, so it provides more power for studying risk and protective factors in a lockdown situation. Second, we were able to control a wide range of possible covariates from sociodemographic and socioeconomic characteristics of children's families to children's schooling. To our knowledge, this is the first study to consider a set of variables related to children's housing and schooling**.** Third**,** using validated measures of hyperactivity/inattention and emotional symptoms (SDQ) with satisfactory psychometric properties is a strength and novel aspect of this study^31,34^. Overall, we believe that our findings contribute to the evolving debate concerning school closure and children's mental health. We suggest that school closure due to COVID-19 could increase the risk of mental health problems and exacerbate health inequalities^71^. Our findings provide important guidance for the development of children's psychological support strategies in France.

### Implications

The current focus on measures to contain COVID-19 transmission around the world may distract attention from emerging mental health issues^57,80^. The closure of schools due to COVID-19 and the lockdown could lead to an increase in mental disorders in children, especially among vulnerable populations. Efforts must be made to address the pandemic situation while considering children's mental health and to adapt the delivery of care to meet the demands of COVID-19^71^. Our findings have important clinical and policy implications. In this global public health emergency, identifying vulnerable groups of children and environmental risk factors of children's mental health is a critical step in developing and adapting mental health services and tools^81^. Health authorities and governments should implement services that improve the mental health of the population, particularly that of vulnerable children. Emerging digital applications such as telehealth, social media, and mobile health could serve to promote children's mental health^82^.

## Conclusion

This study conducted in France explored several risk factors of hyperactivity/inattention and emotional symptoms in children during the school closure due to the COVID-19 pandemic. Some factors that may increase the risk of children developing mental health problems are described: sex, pre-existing health disorders, COVID cases in the household, difficulties in sleeping and in schooling, and sociodemographic and socioeconomic disparities. Policymakers need to balance the pros and cons of reopening school, taking into consideration the educational and psychological consequences for children. Therefore, it is essential to implement appropriate protective measures to manage the unprecedented repercussions of school closure on children's mental health while avoiding an increase in socio-economic inequalities.

## Supplementary Information

Supplementary Information.

## Data Availability

In regards to data availability, data of the study are protected under the protection of health data regulation set by the French National Commission on Informatics and Liberty (Commission Nationale de l’Informatique et des Libertés, CNIL). The data can be available upon reasonable request after a consultation with the steering committee of the Sapris study. The French law forbids us to provide free access to Sapris data; access could however be given by the steering committee after legal verification of the use of the data.

## Abbreviations

ADHD: Attention-deficit/hyperactivity disorder
aOR: Adjusted odds ratio
CART: Classification and regression tree
CI: Confidence intervals
OR: Odds ratio
SDQ: Strength and difficulties questionnaire
VIP: Variable inclusion probability
