# Supplementary material for: Children's mental and behavioral health, schooling, and socioeconomic characteristics during school closure in France due to COVID-19: the SAPRIS project
Source: Sci Rep. 2021 Nov 17;11:22373. doi: 10.1038/s41598-021-01676-7 (PMC8599695; doi:10.1038/s41598-021-01676-7)
Supplement: Supplementary file 1 — Supplementary Information. [file 41598_2021_1676_MOESM1_ESM.docx]

**ONLINE SUPPLEMENTS**

**Weighting**

For ELFE data, a weighting coefficient was calculated from six calibration variables, six commonly used attrition variables and five variables specific to the SAPRIS study. The calibration variables are the region of residence, whether the mother was primiparous, the marital status, mother's age, the mother's level of education and her immigrant status. The six attrition variables were the presence of childbirth preparation sessions, the activity of the father and mother at the time of delivery, the father's age, the mother’s relationship status at birth, alcohol consumption during pregnancy and twin birth. In addition, a SAPRIS-specific weighting also took into account the child’s sex, housing type, the area of residence, the family situation and the region of usual residence.

For EPIPAGE-2 data, inverse probability weighting was used to correct for non-representativeness: non-participation bias among children surviving at the time of the SAPRIS survey and to take into account the differences in the recruitment periods in survey design between gestational age groups (8 months for infants born at 24-26 weeks, 6 months for 27-31 weeks and 5 weeks for 32-34 weeks). Non-participation weights were estimated using logistic regression models, with participation as the response variable, and socio-economic and perinatal factors (birth region of child, gestational age, multiple pregnancy, maternal age at birth, maternal birth country, living in a couple relationship at birth, maternal employment status during pregnancy, maternal education level, primiparity, children sex, severe neonatal morbidities) as covariates.
